# Personal

**Published:** 1901-03

**Authors:** 


### PERSONAL.

Dr. Lucien Howe, of Buffalo, was awarded a prize at the recent annual meeting of the Medical Society of the State of New York, for an essay entitled, "On the measurement of the intraocular base line and the size of the meter angle." The fund for this prize was bequeathed by Merritt H. Cash, a former member of the society. The accumulated value, about $100, is to be given in the form of a medal, if desired.

---

Professor Joseph McFarland, M. D., of Philadelphia, a recognised writer, teacher and authority on bacteriology, has removed to Detroit to engage in scientific research in the biological laboratories of Messrs. Parke, Davis & Co. Professor McFarland will thus become an associate of Drs. Charles T. McClintock and E. M. Houghton in the important field of biology and bacteriology.

---

Dr. James T. Burdick of, Brooklyn, has been appointed Surgeon of the New York Soldiers' and Sailors' Home at Bath, vice O. W. Smith, resigned. It is reported, however, that Dr. Smith recalled his resignation before it was acted upon, and hence will contest the action of the trustees in appointing his successor.

---

Dr. P. W. Van Peyma, of Buffalo, has been elected chairman of the Board of School Examiners for the year 1901. Dr. Van Peyma's great interest in the schools and his untiring energy in their behalf is ample justification for this action of the board.

---

Dr. W. Scott Renner, of Buffalo, will sail for Europe, March 6, 1901. He will spend about two months in Berlin in the further study of the specialty with which he has been successfully identified for several years. He will return about May 20th.

---

Dr. J. Hilton Waterman, of New York, has been elected secretary of the Harvard Medical Society of New York City for the year 1901. Dr. Waterman's many Buffalo friends will be pleased to learn of his preferment to this post.

---

Dr. Matthew D. Mann, of Buffalo, has been appointed Park Commissioner of this city in place of Mr. C. R. Huntley, resigned. Dr. Mann's commission holds until May, 1902.

---

Dr. Dewitt G. Wilcox, of Buffalo, was elected secretary of the Homeopathic Medical Society of the State of New York, at its recent annual meeting held at Albany.

---

Dr. Harriet Watson Noble, of Albion, N. Y., has been appointed resident physician at the Western House of Refuge for Women, which is located in that village.

---

Dr. V. M. Griswold, U. of B. '80, who has resided and practised medicine for many years at Fredonia, N. Y., has removed to Westerly, Rhode Island.

---

Dr. Julius Ullman, of Buffalo, has been appointed physician to Buffalo University Dispensary in the department of internal medicine.

---

Dr. and Mrs. Matthew D. Mann, of Allen street, entertained a group of physicians at dinner just before the Lenten season began.

---

Dr. O. C. Shaw, U. of B. '73, has removed from Hamburg, N. Y., to Cassadaga, N. Y.

